# The effect of environmental factors on the genetic differentiation of *Cucurbita ficifolia* populations based on whole-genome resequencing

**DOI:** 10.1186/s12870-023-04602-3

**Published:** 2023-12-15

**Authors:** Shuilian He, Gengyun Li, Jing Zhang, Yumei Ding, Hongzhi Wu, Junjun Xie, Hang Wu, Zhengan Yang

**Affiliations:** 1 College of Landscape and Horticulture, Yunnan Agricultural University, Kunming, 650201 Yunnan China; 2 Key Laboratory of Vegetable Biology of Yunnan Province, College of Landscape and Horticulture, Yunnan Agricultural University, Kunming, 650201 Yunnan China; 3 College of Food Science and Technology, Yunnan Agricultural University, Kunming, 650201 Yunnan China

**Keywords:** *Cucurbita ficifolia*, Environmental factors, Genetic diversity, Genome re-sequencing, Population genetics

## Abstract

**Background:**

*Cucurbita ficifolia* is one of the squash species most resistant to fungal pathogens, and has especially high resistance to melon Fusarium wilt. This species is therefore an important germplasm resource for the breeding of squash and melon cultivars.

**Results:**

Whole-genome resequencing of 223 individuals from 32 populations in Yunnan Province, the main cucurbit production area in China, was performed and 3,855,120 single-nucleotide polymorphisms (SNPs) and 1,361,000 InDels were obtained. SNP analysis suggested that levels of genetic diversity in *C. ficifolia* were high, but that different populations showed no significant genetic differentiation or geographical structure, and that individual *C. ficifolia* plants with fruit rinds of a similar color did not form independent clusters. A Mantel test conducted in combination with geographical distance and environmental factors suggested that genetic distance was not correlated with geographical distance, but had a significant correlation with environmental distance. Further associations between the genetic data and five environmental factors were analyzed using whole-genome association analysis. SNPs associated with each environmental factor were investigated and genes 250 kb upstream and downstream from associated SNPs were annotated. Overall, 15 marker-trait-associated SNPs (MTAs) and 293 genes under environmental selection were identified. The identified genes were involved in cell membrane lipid metabolism, macromolecular complexes, catalytic activity and other related aspects. Ecological niche modeling was used to simulate the distribution of *C. ficifolia* across time, from the present and into the future. We found that the area suitable for *C. ficifolia* changed with the changing climate in different periods.

**Conclusions:**

Resequencing of the *C. ficifolia* accessions has allowed identification of genetic markers, such as SNPs and InDels. The SNPs identified in this study suggest that environmental factors mediated the formation of the population structure of *C. ficifolia* in China. These SNPs and Indels might also contribute to the variation in important pathways of genes for important agronomic traits such as yield, disease resistance and stress tolerance. Moreover, the genome resequencing data and the genetic markers identified from 223 accessions provide insight into the genetic variation of the *C. ficifolia* germplasm and will facilitate a broad range of genetic studies.

**Supplementary Information:**

The online version contains supplementary material available at 10.1186/s12870-023-04602-3.

## Background

A common topic in ecological genetics is the effect of a changing environment on the distribution and composition of genetically variable populations [1], as well as the mechanisms by which the genetic structure of populations may evolve and diverge [2]. Climatic factors are known to be important for the distribution of plants and are important drivers of evolution [3]. Plant populations do not remain genetically identical over time, and different populations of a species in different habitats or areas may be genetically distinct, adapting genetically and physiologically to local conditions [4]. Small-scale patterns in genetic structure are thus often driven by environmental factors [5, 6]. The effects of the environment can sometimes even be observed in populations of a species with a high potential for gene flow [2, 7].

*Curcubita ficifolia* Bouché (Cucurbitaceae) is a short-day plant, is sensitive to temperature and is not heat-resistant. The plant is monoecious and has unisexual flowers. It is known as “black seed squash” in English after its black seeds, and the Chinese name translates "rice noodle squash" or "Black Seeded’ figleaf squash" in Chinese [8] as the fruit pulp resembles rice noodles or vermicelli. *C. ficifolia* is a trailing herb with perennial roots. In sunny, warm environments, the plant grows as a perennial, but in areas that get severe winter frosts, the roots die off over winter and the plant grows as an annual. New vines can grow as much as 20 m in a year, and more than 50 high-yield squashes can be produced per plant in this time [9]. A single fruit generally weighs 2 to 5 kg and produces 50 to 150 g of seeds. The younger, tender fruits can be eaten by humans as a vegetable, whereas the slightly older, larger fruits are lignified, and are generally fed to livestock. The shells of older squashes are hard, and the fruits store well. The rind is found in three color patterns: green (dark green alternating with light green), white and “decorative” (green alternating with white) [10]. The ripe seeds are black or dark brown and also have important edible value. Compared with other species of Cucurbitaceae, *C. ficifolia* has strong resistance to a number of environmental stresses, including cold and drought, it grows well on barren soils and is also resistant to several diseases. The species also shows a particularly high resistance to melon Fusarium wilt and is therefore an important germplasm resource for the breeding of new cucurbit cultivars.

To date, there have been few studies investigating *C. ficifolia*. Some studies have focused on its pharmacological effects, and *C. ficifolia* extract has been found to increase the secretion of insulin, playing an important role in lowering blood sugar and can therefore be helpful in the treatment of diabetes [11–14]. *C. ficifolia* extracts can also modulate systemic chronic inflammation in obese mice, where they may have beneficial effects on the adaptive immune system [15]. Other studies concern the use of *C. ficifolia* as a rootstock onto which other species of *Cucurbita* can be grafted, and have found that *C. ficifolia* rootstocks confer improved yields and enhanced resistances to cold, salt and diseases including Fusarium wilt [10, 16–19]. Cucumbers grafted onto *C. ficifolia* rootstock and grown in plastic greenhouses have increased foreign income from fresh produce in Liaoning, Shandong and several other provinces in China, by virtue of the early market supply and high quality of the grafted plants. Moreover, *C. ficifolia* has become an excellent germplasm resource for improving the disease resistance in new cultivars. Brazil exports the largest volume of *C. ficifolia* worldwide, and Japan imports the most [8].

*C. ficifolia* originated in Central and South America [20], and the wild plant is common in mountainous areas from northern Mexico to northern Argentina and Chile. However, it is now cultivated in many countries worldwide, and has been popular for many centuries in Asia [21]. There is no data on the history of introduction and cultivation of *C. ficifolia* in China, but the species has a long history of cultivation in Yunnan, Sichuan, Guizhou and other higher altitude regions [22]. *C. ficifolia* grows well at high altitudes*,* and much of Yunnan Province is thus suitable for its cultivation, with this province becoming the main production area in China. *C. ficifolia* is an important breeding germplasm resource for cucurbits, however, to date there have been only a few genetic studies investigating this species. In order to more effectively utilize and develop this species as a genetic resource, a better understanding of its genetic diversity, the extent by which its populations differ genetically and the factors underlying these patterns are required.

Whole genome resequencing and genome-wide association studies (GWAS) have been used to investigate the genomes of several widely grown cucurbit crops, including *Cucurbita pepos*, pumpkins, cucumbers, watermelons and melons, and many SNPs controlling horticulturally important phenotypes have been discovered [23–28]. However, despite the utility and potential of *C. ficifolia* in agriculture, the *C. ficifolia* genome has not to date been studied in depth. In this project, we investigated genetic variation in different *C. ficifolia* populations in Yunnan using whole-genome resequencing. The main aims of the study were: (1) to use whole-genome resequencing to obtain a library of SNP markers, and to evaluate genetic diversity in *C. ficifolia* in Yunnan, (2) to use population genetic approaches to elucidate the genetic structure of *C. ficifolia* within and among populations, as well as to investigate the evolutionary patterns behind rind of different colors, and (3) to analyze how geographical and environmental distances affect the genetic structure of *C. ficifolia*. The data will improve the utilization and development of *C. ficifolia* germplasm resources.

## Results

### Plant materials and phenotypic statistics

**Fig. 1:**
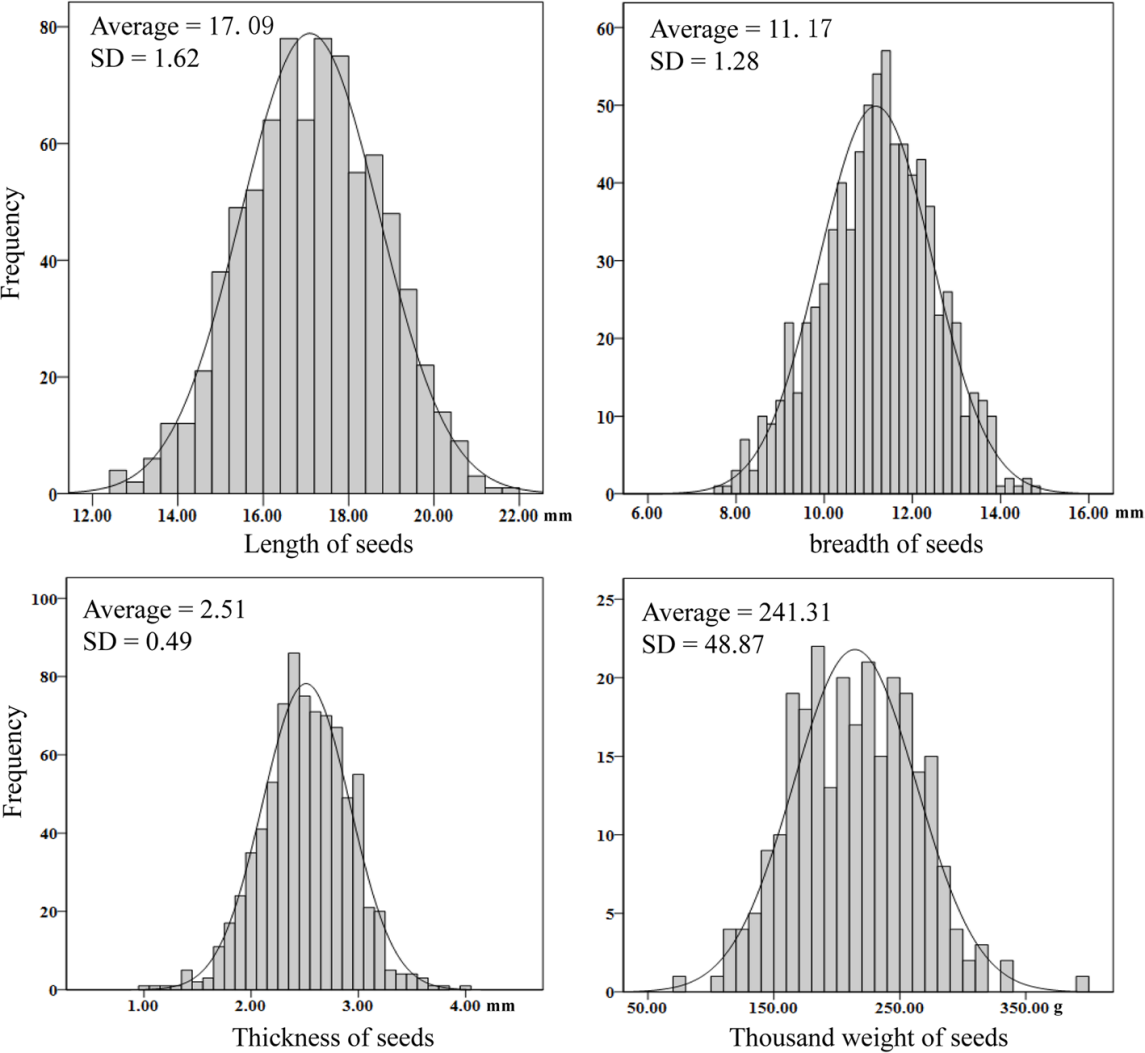
Normal distribution of the length, breath, thickness and thousand weight of *Cucurbita ficifolia* seeds

Two hundred twenty-three individuals were collected from the field in Yunnan province of China. In order to avoid the homogenization of research materials, phenotypic traits were recorded and analyzed for all the samples. The length, breadth, and thickness of the seeds, the thousand seed weight were analyzed for each sample (Fig. 1 and Table S1). The average length of seed is 17.09 mm, beath is 11.07 mm, thickness is 2.51 mm and thousand weight is 241.31. All four phenotypic indices conform to a normal distribution. We also measured the squash rind color and showed that 109 individuals of which were ‘decorative’; 57 were white and 30 were green. We can see that the phenotypic diversity of our samples was high and suitable for genetic analyses as natural populations.SNP Calling and Analyses of Genetic Diversity and Linkage Disequilibrium Decay.

**Fig. 2:**
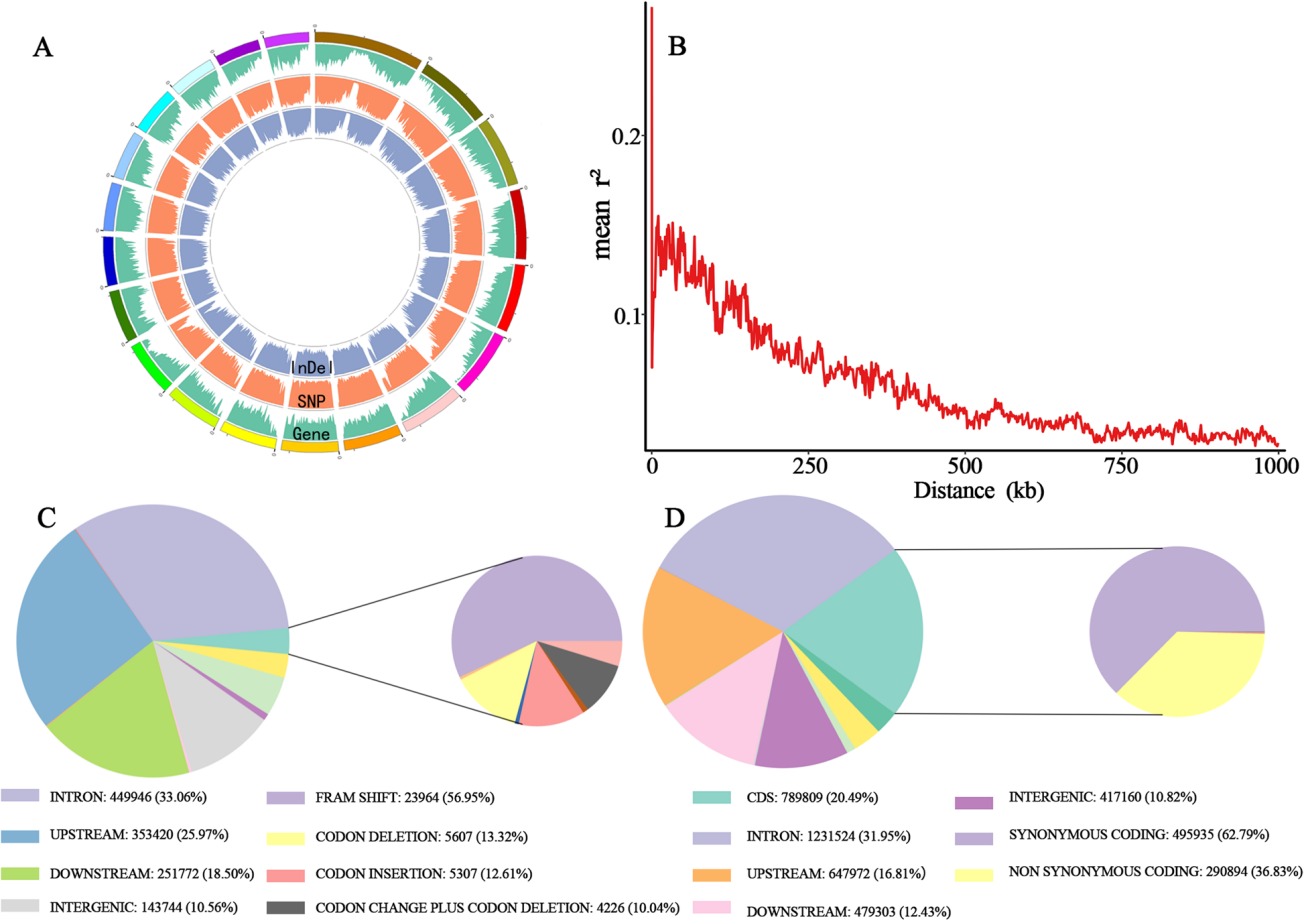
Genome-wide variations among 223 *Cucurbita ficifolia* samples. **A** Circus diagram of the 20 predicted pseudomolecules of *Cucurbita ficifolia* illustrating the density distributions of InDels (blue inner circle), SNPs (orange middle layer) and genes (green outer circle) within the 223 *Cucurbita ficifolia* samples. **B** Plots of squared allele frequency correlations (*r*^2^) by physical distance between sites in *Cucurbita ficifolia.* **C** SNP annotation classification results.** D** InDels annotation classification results

Resequencing the 223 *C. ficifolia* individuals on an Illumina NovaSeq6000-sequencer generated 758.08 Gbp of clean data, with a total of 2.2 million 100 bp paired-end reads (332 Gb of sequence data), 93.63% of which had Q values ≥ 30. The average alignment rate of samples with the reference genome was 93.17%, the average depth of coverage was 9 × and the genome coverage was 66.11% (with at least one base coverage).There is, to date, no published genome information available for *C. ficifolia*. We mapped our resequencing data to whole genomes of four species of *Cucurbita* (*C. argyrosperma* subsp. *Sororia*; *C. pepo* subsp. *pepo*; *C. moschata*; *C. maxima*)*.* The mapping rate to the *C. moschata* genome was found to be the highest (93.88%), and the *C. moschata* genome was therefore selected as the reference genome for this study. After mapping to the *C. moschata* reference genome [29], we identified 3,855,120 SNPs and 1,361,000 InDels shorter than or equal to 6 bp (Table S2). The genome-wide variations among the 223 *C. ficifolia* samples are summarized in Fig. 2. We constructed 20 pseudochromosomes, using the *C. moschata* genome as a reference. The densities of the genes, SNPs and InDels were evenly distributed over all 20 pseudochromosomes (Fig. 2A).

We calculated *r*^2^ between pairs of SNPs to investigate patterns of linkage disequilibrium between the different *C. ficifolia* chromosomes. At 250 kb, the linkage disequilibrium across all samples dropped to half its maximum (*r*^2^ = 0.08, Fig. 2B). Of the 3.86 million SNPs, 47.56% were present in intergenic regions and 52.44% were located intragenically (CDS: 20.49%; intron: 31.95) (Fig. 2C). Of the 1.36 million InDels, most (63.85%) were present in intergenic regions and the intragenic InDels were predominantly introns (CDS: 3.09%; intron: 33.06%) (Fig. 2D).

To understand the extent of genetic diversity in *C. ficifolia*, we calculated the number of polymorphic markers and tested five common genetic diversity indices. The average number of polymorphic sites in each population was 26,376 (between 17,151 and 33,012), and the genetic diversity of *C. ficifolia* was high, with a π of 0.354 (0.252–0.418). The expected heterozygosity (*H*_*E*_) was 0.319 (0.221–0.404), the observed heterozygosity (*H*_*O*_) was 0.392 (0.315–0.466), the polymorphism information content (PIC) was 0.251 (0.173–0.316) and the Shannon Wiener index (*I*) was 0.465 (0.320–0.587) (Table 1). Of the 32 populations sampled, JS had the highest genetic diversity (π = 0.418), followed by DL and DY (π = 0.417). This high genetic diversity could provide abundant genetic information for cucurbit crop breeding.

**Table 1:** Genetic diversity indexes of *Cucurbiba ficifolia* populations

Population	Number of individuals	Number of polymorphic markers	π	*H*_*E*_	*H*_*O*_	PIC	*I*
DC	5	21,948	0.284	0.254	0.371	0.200	0.373
DL	4	29,854	0.417	0.373	0.343	0.291	0.540
DY	2	21,516	0.417	0.313	0.465	0.243	0.449
FM	3	26,664	0.414	0.342	0.418	0.267	0.494
FQ	5	26,856	0.367	0.329	0.420	0.257	0.478
FY	4	26,973	0.382	0.332	0.391	0.260	0.483
GJ	15	32,910	0.405	0.391	0.425	0.307	0.571
GM	5	29,366	0.394	0.352	0.361	0.278	0.515
GN	5	26,647	0.356	0.319	0.397	0.251	0.466
HP	4	23,483	0.322	0.280	0.375	0.221	0.410
HQ	6	19,646	0.260	0.237	0.340	0.186	0.345
HZ	12	32,487	0.411	0.394	0.439	0.308	0.572
JD	3	17,151	0.268	0.221	0.340	0.173	0.320
JG	17	31,181	0.402	0.372	0.404	0.292	0.543
JS	16	33,012	0.418	0.404	0.466	0.316	0.587
LQ	8	30,786	0.381	0.356	0.431	0.281	0.523
LY	3	17,224	0.270	0.224	0.348	0.174	0.322
MLP	6	22,831	0.305	0.278	0.349	0.218	0.405
MLQ	8	32,044	0.407	0.380	0.390	0.299	0.556
MLS	6	24,959	0.301	0.274	0.358	0.218	0.408
NH	19	32,981	0.396	0.385	0.426	0.304	0.565
NJ	5	24,855	0.339	0.303	0.348	0.237	0.440
PLQ	3	25,187	0.385	0.318	0.438	0.249	0.461
SP	15	32,727	0.409	0.395	0.465	0.310	0.575
TC	5	19,516	0.270	0.242	0.368	0.189	0.350
XY	5	20,166	0.273	0.244	0.370	0.191	0.355
YA	6	30,780	0.399	0.364	0.412	0.287	0.534
YB	5	18,681	0.252	0.225	0.315	0.177	0.328
YL	10	32,242	0.407	0.386	0.447	0.303	0.563
YM	12	31,820	0.377	0.361	0.423	0.285	0.532
ZX	5	28,671	0.383	0.342	0.374	0.269	0.500
ZY	5	18,883	0.255	0.227	0.318	0.178	0.332
Total	232	26,376	0.353	0.319	0.392	0.251	0.465

*π* Nei’s genetic diversity, *H*_*E*_ expected heterozygosity, *H*_*O*_ Observed heterozygosity, *PIC* Polymorphism information content, *I* Shannon Wiener index

### Genetic differentiation, phylogeny construction and population structure analysis

**Fig. 3:**
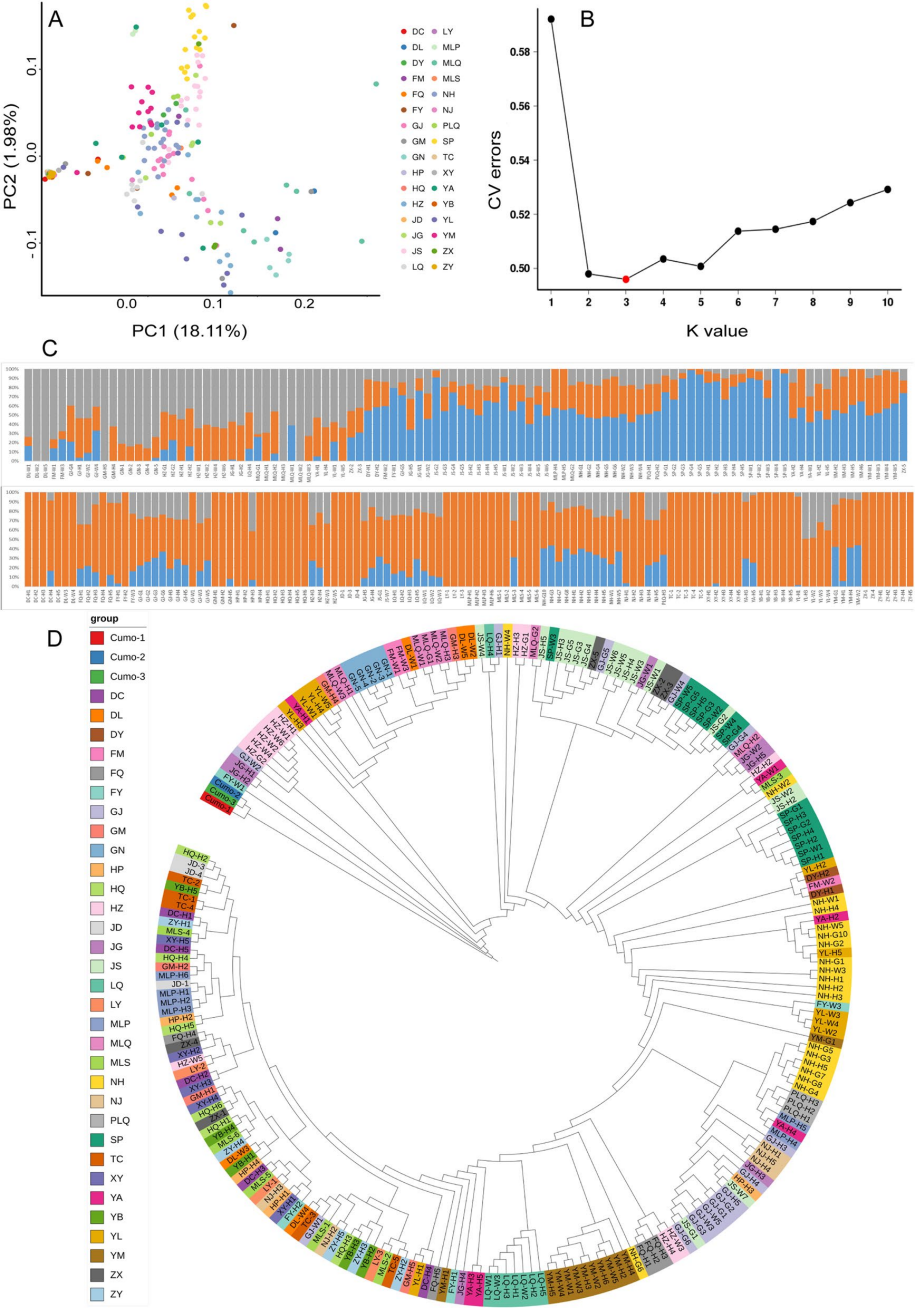
Analysis of phylogeny and population structure in *C. ficifolia*.** A** Population structure within the diversity panel assessed using principal component analysis. **B** Plot of different number of subpopulations (*K*) from 1 to 10 versus cross validation error (CV error). The cross validation procedure as implemented in ADMIXTURE was used to initially find the optimum number of subpopulations (*K*) by minimizing the cross validation error. **C** Population structure based on *K* = 3. The different samples are shown on the x-axis. The y-axis quantifies the membership probability of samples belonging to different groups. Colors in each row represent structural components. **D** Neighbor-joining (NJ) phylogenetic tree

Our Arlequin analysis suggested that the Wright's *F*_ST_ value among populations was 0.03, indicating that the degree of genetic differentiation among different *C. ficifolia* populations was very small. The pairwise *F*_ST_ values of each population ranged from 0 to 0.841 (Table S3), showing that genetic distances among populations varied greatly. We then used a combined approach (PCA, maximum likelihood estimation of individual ancestries, and an NJ phylogenetic tree) to analyze the phylogenetic and population genetic patterns present in *C. ficifolia* populations. The PCA analysis reflected the larger trend, and suggested that the genetic makeup of individuals in the same population were usually relatively similar, however, the population did not form distinct groups (Fig. 3A). We then applied ADMIXTURE to make a maximum likelihood estimation of the individual ancestries of the samples. In this analysis, the range of *K* = 1-10 was analyzed, and the cross-validation (CV) errors value reached the lowest value at *K* = 3, indicating that the optimal grouping of *C. ficifolia* was 3 (Fig. 3B). These assignments were not completely separated by geography, but there was a geographical trend. Most of the individuals in Cluster I were from the populations in eastern Yunnan, those in Cluster II were mostly from the populations in central Yunnan, and those in Cluster III were from populations in western and northeastern Yunnan or other regions. There were also some populations whose individuals were divided between three clusters, such as ZX, YA and others (Fig. 3C). The reconstructed phylogenetic tree suggested that most individuals from the same populations were closely related, for example in populations NH, JS and SP. However, no clear clustering by geographic distribution was apparent (Fig. 3D).

The rind of *C. ficifolia* fruits can be one of three colors: green (dark green alternating with light green, -G); white (-W); and decorative (green alternating with white, -H). The three rind colors did not form independent clusters in either the reconstructed phylogenetic tree or in our genetic structure analysis. However, individuals from the same local population, regardless of the color of the rind, tended to cluster in the same branch, indicating that individuals of different colors within the local population were closely related, while individuals with the same rind color from different regions were less related.

Potential correlations between genetic distance and geographic and environmental distance were tested using Mantel tests. No significant correlation between genetic and geographic distances was found (*r* = 0.007; *P* = 0.176), although there was a more obvious relationship between genetic and environmental distances (*r* = 0.125; *P* = 0.047). In order to exclude interference from environmental or geographical factors, partial Mantel tests were conducted. These tests agreed with the simple Mantel tests, and showed no correlation between genetic and geographic distance (*r* = 0.220; *P* = 0.386); when we controlled for geographical distance, genetic distance and environmental distance were still correlated (*r* = 0.106; *P* = 0.043) (Table 2). The above analyses strongly indicate that the genetic structure of *C. ficifolia* is driven by environmental factors.

**Table 2:** Correlations between genetic, geographic and environmental distances as tested using Mantel tests and partial Mantel tests

	Mantel Test		Partial Mantel Test	
	*r*	*P* value	*r*	*P* value
Gen & Geo	0.007	0.176	0.220	0.386
Gen & Env	0.125	**0.047**	0.106	**0.043**

Regular type refers to non-significant results and bold type refers to significant correlations. *Geo *geographical distance, *Gen *genetic distance, *Env *environmental distance

### Marker-trait association analyses

**Fig. 4:**
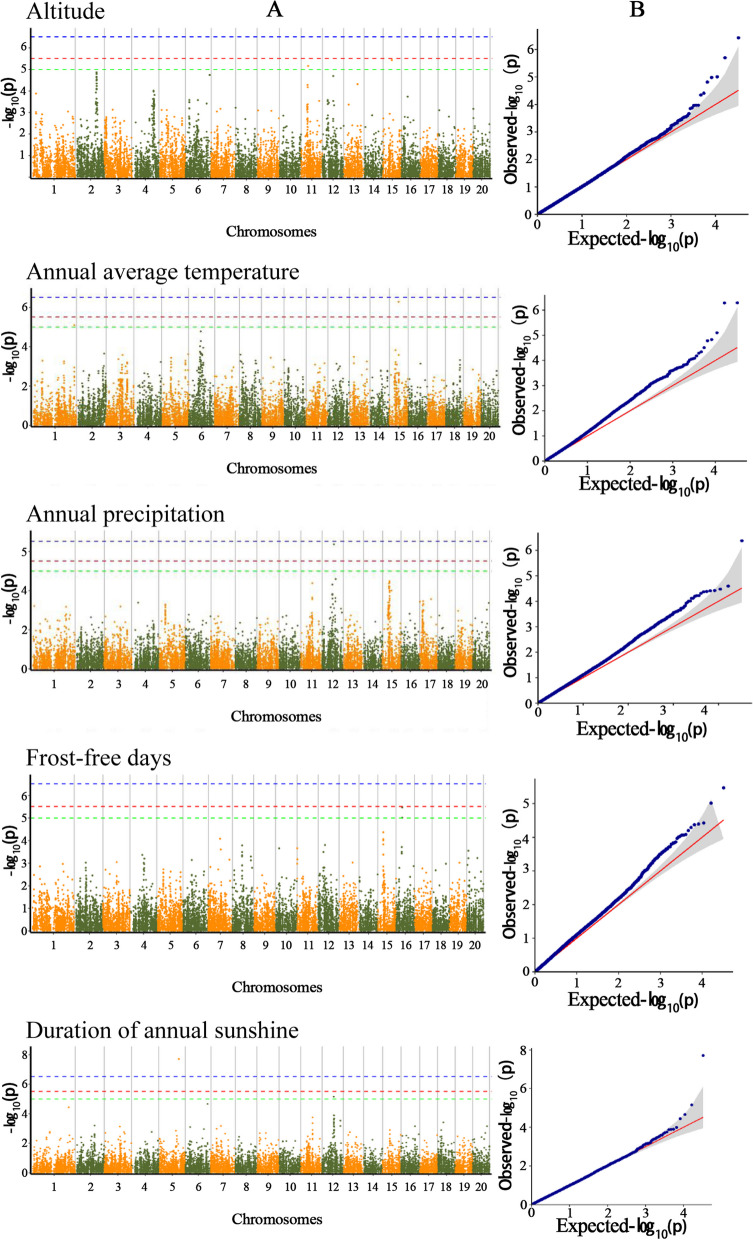
Manhattan and QQ plots showing results from genome-wide association mapping of marker-trait association analyses. **A** GEMMA model Manhattan maps. The x-axis of each plot represents the physical position of each SNP, and the y-axis displays the negative log10 of *p*-values for each SNP included in the GWAS. **B** GEMMA model quantile–quantile plots (QQ plots)

We used 3.85 million SNPs together with climate data (including annual mean temperature, annual precipitation, altitude, frost-free season and annual average sunshine) from the 32 sampling regions in an association analysis in order to identify any SNP sites associated with climate traits. We used several different statistical programs and simulations for this analysis, and performed comparisons in GEMMA (LM and LMM models), FaST-lmm, and EMMAX. The results varied to some extent with the different statistical models. The LM model of GEMMA found the greatest number of relevant marker-trait associated SNPs. Taking log_10_(*p*) = 5 as the reference point, the five environmental factors were associated with a total of 15 MTAs (Fig. 4), using the all the four models. Two MTAs were associated with altitude (T/C in chromosome NW_527.1 and C/A in NW_531.1); two MTAs were associated with annual average temperature (G/T in chromosome NW-531.1 and C/T in chromosome 517.1); one MTAs was associated with annual precipitation (A/G in chromosome NW-528.1); eight MTAs were associated with duration of annual sunshine (T/G in chromosome NW_521.1; A/T in chromosome NW-534.1; five SNP in chromosome NW_528.1) and two MTAs were associated with frost-free days (C/T and G/A in NW_532.1) (Table 3). The number of MTAs identified showed that duration of annual sunshine had the greatest impact on genetic variation in *C. ficifolia*.

**Table 3:** Marker-trait-associated SNPs with climate factors

Trait	chromosome	Position	*P* value	-log10*P*	Allele	Count	MAF
Altitude	NW_019268527.1	1,819,951	6.93E-06	5.16	T/C	T:185_C:28	0.13
Altitude	NW_019268531.1	2,314,442	3.67E-06	5.44	C/A	C:194_A:26	0.12
Annual average temperature	NW_019268531.1	2,389,878	5.16E-07	6.29	G/T	G:38_T:175	0.18
Annual average temperature	NW_019268517.1	10,717,495	7.94E-06	5.1	C/T	C:173_T:23	0.12
Annual precipitation	NW_019268528.1	3,116,384	4.25E-07	6.37	A/G	A:141_G:75	0.35
Duration of annual sunshine	NW_019268521.1	5,309,956	1.58E-08	7.8	T/G	T:183_G:40	0.18
Duration of annual sunshine	NW_019268534.1	1,535,409	5.02E-06	5.3	A/T	A:64_T:155	0.29
Duration of annual sunshine	NW_019268528.1	3,065,008	4.70E-08	7.33	C/A	C:137_A:76	0.36
Duration of annual sunshine	NW_019268528.1	3,083,925	5.18E-06	5.29	G/C	G:170_C:51	0.23
Duration of annual sunshine	NW_019268528.1	3,086,479	7.96E-06	5.1	A/G	A:48_G:120	0.22
Duration of annual sunshine	NW_019268528.1	3,089,068	4.59E-06	5.34	G/A	G:173_A:49	0.22
Duration of annual sunshine	NW_019268528.1	3,095,958	7.89E-06	5.1	C/A	C:166_A:45	0.21
Duration of annual sunshine	NW_019268528.1	3,101,942	6.25E-06	5.2	T/G	T:47_G:167	0.22
Frost-free days	NW_019268532.1	1,633,356	9.68E-06	5.01	C/T	C:21_T:186	0.1
Frost-free days	NW_019268532.1	1,641,919	3.38E-06	5.47	G/A	G:38_A:181	0.17

*MAF* Minor allele frequency

A total of 293 genes within 250 kb of these MTAs (including both up- and downstream genes) were identified (Table S4). An enrichment analysis of the annotated genes showed that the up- and downstream genes of MTAs were involved in the following three functions: “cellular component”, “molecular function” and “biological process” (Fig. S1).These genes reflected the changes in *C. ficifolia* cell activity, molecular function and biological processes in response to different environmental conditions.Some genes, including “gene control cellular component: integral component of membrane” (GO: 0016021), “mitochondrial inner membrane” (GO: 0005743), and “spindle microtubule” (GO: 0005876) were revealed when we annotated the MTAs with duration of annual sunshine. This indicated that different durations of annual sunshine have an effect on the cell structure of *C. ficifolia* in different populations.

### Present and future distributions of *C. ficifolia*

**Fig. 5:**
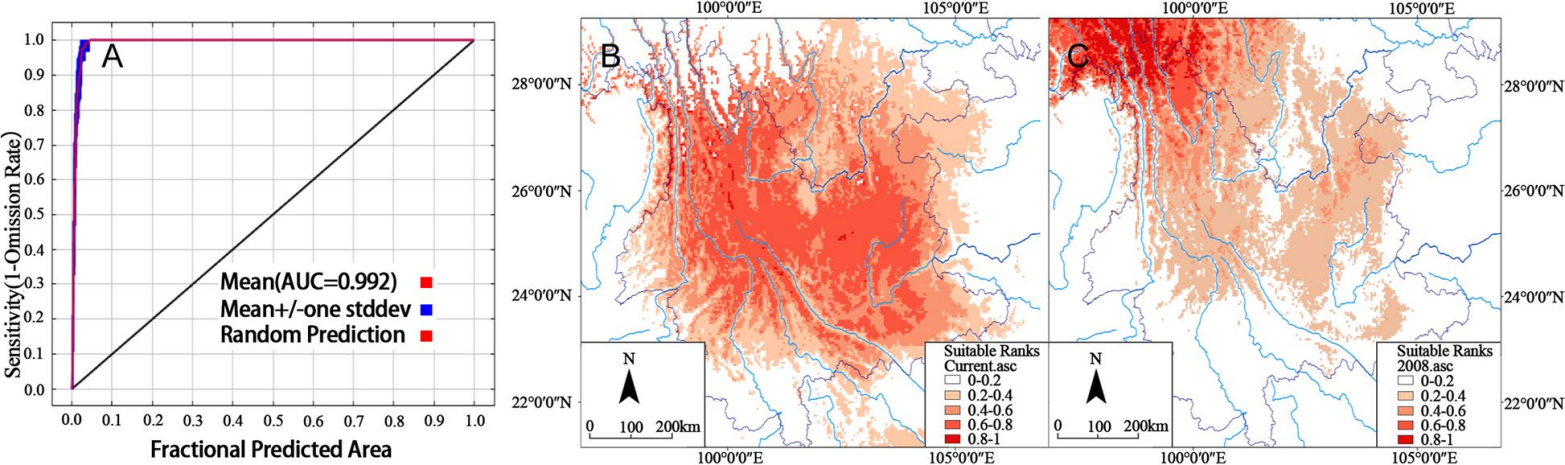
**A** Reliability analysis of model prediction;** B** present and (**C**) predicted future distributions of *Cucurbita ficifolia.* Each cell on the grid map has a suitability index between 0 and 1. Low values (white or pale red) indicate that conditions are unsuitable for the species to occur, whereas high values (dark red) indicate that conditions are suitable. Note: The MAP is taken from CGIAR-CSI (https://srtm.csi.cgiar.org)

In order to further confirm the influence of environmental factors on the distribution of *C. ficifolia*, we performed ecological niche modeling to simulate the distribution of *C. ficifolia* during the present distribution, and that in the future (2080). All models performed well, with AUC values > 0.9 (*n* = 10 replicate model runs) [30] (Fig. 5A). *C. ficifolia* prefers a cooler environment, with a long growth period and fruit maturation in between November and January period. If it is too cold in winter, the fruit does not normally produce seed. Several years of data verification and field investigations suggest that the *C. ficifolia* populations in China are currently found mainly distributed in Yunnan and the surrounding areas. The distribution of *C. ficifolia* simulated by the ecological niche modeling based on environmental factors (Fig. 5B) was consistent with the current observed “real” distribution of cultivated *C. ficifolia* in Yunnan, which further illustrates the reliability of the model and also suggests that the distribution of this species in Yunnan is indeed limited by environmental factors.

*C. ficifolia* is an excellent germplasm resource for the improvement of melon, squash and gourd crops. From the distribution map predicted for 2080, it can be seen that the suitable cultivation distribution area of *C. ficifolia* will migrate to the northwest, and the most suitable area will then be concentrated in the Hengduan Mountain Range (Fig. 5C). However, most areas in the southwestern regions of Yunnan will no longer be suitable for the cultivation of *C. ficifolia*, so it is particularly vital to collect and protect germplasm resources currently present in these regions.

## Discussion

### Genetic diversity of *C. ficifolia*

The study of genetic diversity provides insight into the origins and evolution, particularly the micro-evolution, of biodiversity. The information generated from these studies can inform animal and plant breeding programs [31]. the genetic structure of populations can be influenced by a number of factors, including the evolutionary history of the populations, their distribution, life form, breeding system and the mechanism of seed dispersal. The combined effect of various factors influences the levels of genetic variation seen within plant populations. Analysis of the spacial structure and genetic variation within plant populations is therefore important when investigating evolutionary factors [31, 32]. High genetic diversity is beneficial for the breeding and improvement of horticultural plants; however, domestication of plants can often lead to reductions in their genetic variability through anthropomorphic selection. This leads to the potential loss of valuable genes, including those conferring resistance and tolerance to biotic and abiotic factors. Although cucurbit crops are playing an increasingly important role in global nutrition, many valuable genes have been lost during domestication and manual selection, and diseases, such as Fusarium wilt, occur frequently [33]. Breeding technology can improve crop yields, disease resistance and adaptability to the environment, and closely related species with high resistances are important genetic resources for this genetic improvement. With the development of high-throughput sequencing technologies, there have been increasing numbers of studies using resequencing to analyze the genetic diversity of agricultural plants. For example, resequenced groups of chickpea resources from around the world were found to have π values ranging from 0.85 to 0.105 [34]. Significant differences in genetic diversity (π) were also found in different groups of soybean (*Glycine soja*, soybean landraces and soybean cultivars) in resequencing studies (*G. soja*: 2.94 × 10^−3^; landraces: 1.40 × 10^−3^; cultivars: 1.05 × 10^−3^) [35]. Similarly, resequencing of gourd resources suggested that wild gourd (*Citrullus colocynthis*, π = 6.75 × 10^−3^) and citron melon (*Citrullus amarus*, π = 2.28 × 10^−3^) had much greater nucleotide diversity than did the watermelons *Citrullus mucosospermus* (π = 0.792 × 10^−3^) and *C. lanatus* (π = 0.56 × 10^−3^) [26, 27]. *C. ficifolia* has relatively high genetic variation, which could provide good germplasm resources for the breeding of novel cucurbit cultivars and the improvement of races. This resequencing study of different local Chinese populations of *C. ficifolia* will provide a good basis for subsequent investigations using GWAS and other means to explore the excellent genetic resources of this species.

### The main factors responsible for the observed genetic structure in *C. ficifolia*

Isolation brought about by geographical distance (IBD) and isolation from environmental distance (IBE) are recognized as important drivers of genetic differentiation [36–39]. Genetic differentiation tends to increase with increasing geographic distances between populations [1]. However, in order to adapt to different environments, the genetic diversity of neighboring populations may be reduced due to natural selection [38], which will promote or maintain genetic differentiation between different environments [40]. The large-scale genetic structure of populations may therefore be influenced by geographic processes, while over smaller scales, the population structure may be influenced more by ecological processes [41]. Climate change over time may also be an important driver of population differentiation [42]. A thorough understanding of the relative contributions of all of these factors is important to fully understand the formation of genetic structure [1, 43, 44].

The Wright's* F*_ST_ value among Chinese populations of *C. ficifolia* was 0.03, indicating that the degree of genetic differentiation among different *C. ficifolia* populations was very small. However, the pairwise *F*_ST_ values between certain populations was extremely high, for example. YA and JD: 0.841 (Table S3). Wright's* F*_ST_ is usually calculated for a large random-breeding population [45]. We therefore think the abnormal value of the *F*_ST_ is likely to be the result of the small numbers of individuals sampled: the JD site comprised only three individuals, and the YA site, six individuals. Furthermore, these nine individuals fell into different clusters in the constructed phylogenetic tree, which is also likely to contribute to this abnormal *F*_ST_ value.

We found that individuals from the same population have a close genetic relationship, however, *C. ficifolia* individuals from different, but nearby, populations are not more closely related than those from far populations. This was confirmed with Mantel and Partial Mantel tests, which also showed that the genetic distance between *C. ficifolia* subpopulations in Yunnan had no correlation to geographical distance. In puzzling contrast to the other species of *Cucurbita*, fruit shape and size in *C. ficifolia* are uniform, and only the rind color differs, with rinds known in three color forms: green, white and “decorative” [10]. Individuals with the same phenotype (rind color) did not cluster together in our genetic diversity or phylogenetic analyses, indicating that the *C. ficifolia* has not formed three distinct subspecies or varieties. Instead, individuals with all three rind colors could be found clustering together according to geographical distribution. This is consistent with Nee et al. (1990) [21], who say a single field anywhere in the range of this species may contain essentially all the variation in fruit morphology known for *C. ficifolia*. Interestingly, the plants with green-rinded fruits have been shown to have greater resistance to Fusarium wilt than those with rinds of other colors [46].

From Mantel and partial Mantel tests, a correlation between genetic and environmental distance was revealed. We speculate that the patterns of genetic structure in *C. ficifolia* occurred mostly due to environmental factors. *C. ficifolia* is a perennial plant, but grows as an annual in cold areas. In Yunnan, China, *C. ficifolia* is perennial, with a long annual growth cycle. The squash matures in December, and in the mild climate of Yunnan, the seeds mature. However, other parts of China are generally too cold in December for natural seed maturity. In other words, the growth and spread of *C. ficifolia* is sensitive to climate. The genetic distances observed between the *C. ficifolia* subpopulations in our study were obviously correlated with environmental distance, which supports our speculation. In order to verify this speculation, ecological niche modeling was used to simulate the distribution of *C. ficifolia* across time, from the present and into the future. The results from the ecological niche modeling suggest that the distribution of *C. ficifolia* will change again in the future. The analysis also suggested that environmental factors mediated the formation of the population structure in this species. As the global climate warms, the range of *C. ficifolia* will move northwards. In addition, because this species is a horticultural plant, human interference and the agricultural use of selected germplasms should also be considered to explain the observed population structure of *C. ficifolia*.

## Materials and methods

### Sampling, DNA extraction and sequencing

**Fig. 6:**
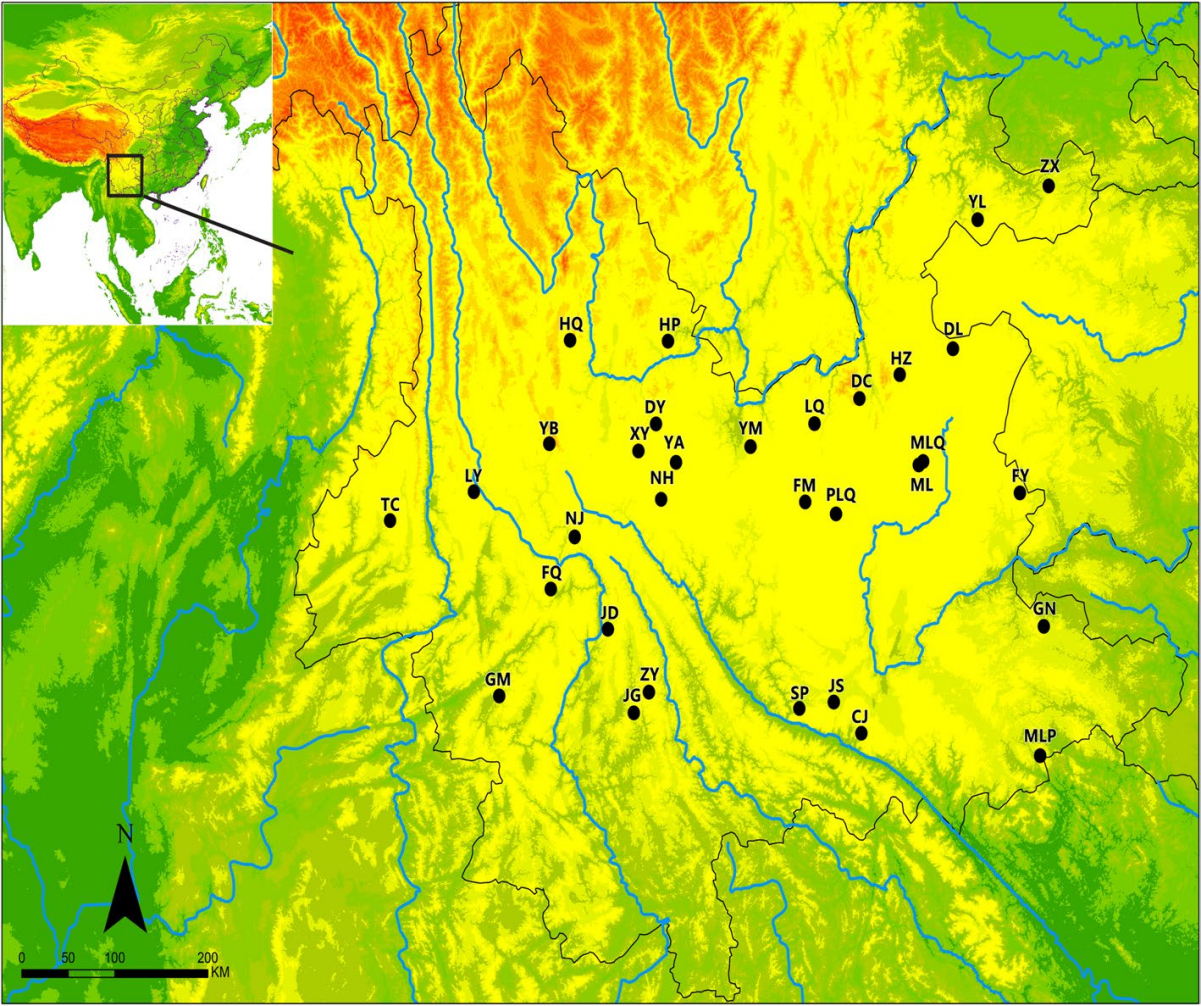
Locations of the 32 sampled *Cucurbita ficifolia* populations in Yunnan. Note: The MAP is taken from CGIAR-CSI (https://srtm.csi.cgiar.org)

The sampling locations were chosen to cover the main *C. ficifolia* production areas in China. Between 3–19 individual plants were sampled from each of 32 locations (Fig. 6 and Table S1), giving a total of 223 *C. ficifolia* samples. Official permits for collection of these native plants were not required because these plants are not included in the list of national key protected plants, and the materials were collected in artificial planting bases or from wild plants in the field. The formal identification of the plant material was performed by Yongjie Guo of Kunming Institute of Botany based on morphological characters. The specimens of *C. ficifolia* has been deposited at Herbarium, Institute of Botany, Chinese Academy of Sciences (voucher # KUN 1580438). All 223 individuals were from landraces and samples were taken from plants at least 50 m apart.The length, breadth, and thickness of the seeds, the thousand seed weight and the squash rind color were recorded for each sample. Fresh leaf samples were stored at -80 °C and total DNA was extracted using the CTAB method [47]. DNA was quantified using a fluorometer with a fluorescent dye (Qubit3.0, Thermo Fisher Scientific, Waltham, MA, United States). DNA quality was assessed using agarose gel electrophoresis (Omega Bio-Tek, Norcross, GA, United States). Each qualified DNA sample was standardized to the same volume (10 µl) and quantity (200 µg) of DNA.

The Illumina NovaSeq6000 sequencing platform was used to randomly fragment the genomic DNA, and the fragments were used to construct libraries of ~ 450 base-pair (bp) inserts and to produce paired-end reads of 150 bp. The raw sequencing data were then filtered using fastp 0.21.0 with the parameters “fastp -q 10 -u 50 -y -g -Y 10 -e 20 -l 100 -b 150 -B 150”. Low-quality reads (where > 50% of the bases had a quality score < 30) and poly-Ns (regions where > 10% of the bases were Ns) were removed. Adapter sequences were trimmed from both ends of the reads and low-quality bases (Q ≤ 13) were removed. The clean reads were then compared with the reference genome *Cucurbita moschata* [29] using the software bwa-mem2 v2.2 [48]. Samtools v1.9 [49] was used to sort the results, after which the sequencing depth, genome coverage and other information from each sample was taken.

### SNP calling, genetic diversity and differentiation analysis

SNPs and InDels were identified using GATK v3.8 [50] with joint calling. Briefly, the genomic variant call format (GVCF) for each sample was obtained using ERC mode from reads where the mapping quality > 20 (parameters were set to “-T Haplotype Caller -ERC GVCF -variant index type LINEAR -variant index parameter 128,000 -mq 20”). Joint variant calling was then conducted using “CombineGVCFs” based on GATK HaplotypeCaller. The variants identified were then hard-filtered to remove low quality calls, with the main filtering parameters set to Q ≥ 30, QD ≥ 2, MQ ≥ 40 and FS ≥ 60. The program vcfutils.pl (varFilter-w 5-W 10) in BCFtools was used to identify SNPs within 10 bp of an adjacent indel, which were then rejected.

The *populations* program in the *Stacks1*.*0* pipeline [51, 52] was used to calculate population genetic statistics (number of private alleles; observed heterozygoisty (*H*_O_); expected heterozygosity (*H*_E_); Nei’s genetic diversity (π)) for each SNP. The pairwise fixation index (*F*_ST_) was calculated using Arlequin [53] to examine the distribution of genetic diversity.

Linkage disequilibrium (LD) decay was calculated between pairs of polymorphic sites using PopLDdecay v3.41 [54] with 3.8 million SNPs. The parameters were set to “-InVCF -OutStat -MaxDist 1000 -MAF 0.0001 -Miss 0.5 -OutType 2”. In order to assess the decay of LD with physical distance, the LD between pairs of polymorphic sites was plotted against genetic distance (bp) between sites in a nonlinear regression. An* r*^*2*^ value = 1 was assumed for a marker distance of 0 kb, and LD decay for each population was evaluated at a cut-off of *r*^2^ = 0.1.

### Reconstruction of phylogeny and analysis of population structure

PLINK51 v1.90 [55] was used with the parameters “-indep-pairphase 100 10 0.2” for all individuals to generate a pruned SNP set (3,855,120 SNPs) for use in the phylogenetic analysis of population structure. Based on the distance matrix, a neighbor-joining tree was then constructed in MEGA X [56] with the parameters “-a *.mao -d *.meg -f mega -o” and the outgroup *Cucurbita moschata*. EIGENSOFT [57] was used with the default parameters to run a principle components analysis and the three components explaining the most variation in the data were identified. ADMIXTURE60 v 1.3.0 [58], (“-C 0.01 -s time -cv -j4” for multiple repeats with different random seeds) was used to make a maximum likelihood estimation of the individual ancestries of the samples.

### Correlations between different genetic, geographical and environmental factors

To investigate any potential relationships between environmental factors and genetic differences in different populations, we performed correlation analyses between the genetic and geographical distances and environmental factors. Environmental distances were calculated in NTSYSPC v2.11c [59] and environmental data were downloaded from http://www.wheata.cn/ [60]. Five key environmental factors affecting the growth and development of *C. ficifolia* were applied in the analyses: annual mean temperature, annual precipitation, altitude, number of frost-free days and annual average sunshine (Table S1). To examine whether genetic distances between pairs of populations increased linearly as a function of geographic or environmental distances, we employed Mantel tests [61] against pairs of distance matrices using the program zt [62], with 10,000 permutations to test for significance.

To determine whether or not the explanation of genetic differentiation by environmental factors was due to isolation, partial Mantel tests were performed using zt [62], again with 10,000 permutations. Matrices of genetic distance, geographical and genetic distances and environmental distance were compared, where appropriate controlling for the effects of environmental or physical distance.

### Marker-trait association analyses

In the IBD test described above, we found that genetic distance was significantly correlated with environmental distance. Based on this, we investigated which of the SNPs responded to environmental factors. We used our resequenced SNPs data and the five key environmental factors mentioned above (annual mean temperature, annual precipitation, latitude, frost-free season and annual average sunshine) from the 32 sampling regions in an association analysis in order to identify any SNP sites associated with climate traits. Each individual in the same population (location) was given the same environmental data, and geographical location, phenotypic information and meteorological factors for each individual were added to the analysis and are shown in Table S4. Association mapping of SNPs with genes was performed in the GEMMA software with the following models: LM and LMM, the generalized linear model (GLM) [63]; factored spectrally transformed linear mixed model (FaST-LMM) [64], and the efficient mixed model association expedited (EMMAX) [63–65]. SNPs were chosen for the association analysis only if their allele frequencies were > 5%. The upper and lower thresholds for screening candidate marker sites were calculated as 0.1 and 0.01. Values within -log10(*p*) = 5 were also fixed as candidate regions. The candidate genes screened using the above thresholds were then functionally annotated. Quantile–quantile plots were used to determine the optimum model, and the linear models were tested by plotting *P* values obtained from the association tests against an expected probability distribution. A cut-off of 0.05 was used to determine significance.

### Ecological niche modeling

In order to further determine the impact of environmental factors on the distribution of *C. ficifolia*, we applied ecological niche modeling (ENM) to simulate the distribution of *C. ficifolia* during the the present and in the future. ENM was performed in MAXENT v3.3.3 [30, 66], as MAXENT can calculate probability distributions using presence-only data from herbarium records [30]. The 32 sampled populations were included in this study (Table S1), and the ENM was performed using the default parameters, except for the following: application of random seed and random test percentage 70%, replicates of 10 and replicated run type bootstrap. Model predictions were visualized in ARCMAP 9.3 (ESRI, Redlands, CA, USA). A general circulation model, CCSM, was used to model the suitability of the future climate for the growth of *C. ficifolia*. In this experiment, we projected the model of climate suitability generated with ecological niche modeling using current climate data onto the global circulation model for the year 2080. The prediction performance analyses were evaluated in MAXENT by calculating the area under the receiver operation characteristic curve.

## Conclusion

The availability of reference genomes and the continuous improvements of genetic data analysis methods are fostering resequencing studies. In this study, we performed the resequencing of *C. ficifolia* accessions, which has allowed identification of genetic markers, such as SNPs and InDels. The SNPs discovered in this study suggest that environmental factors mediated the formation of the population structure of *C. ficifolia*. These SNPs and Indels might contribute to the variation in important pathways of genes for important agronomic traits such as yield, disease resistance and stress tolerance. Moreover, the genome resequencing data and the genetic markers identified from 223 accessions provide insight into the genetic variation of the* C. ficifolia* germplasm and facilitate a broad range of genetic studies.

### Supplementary Information

**Additional file 1: Table S1.** Geographical location, phenotypic information and meteorological factors of sample sites.

**Additional file 2: Table S2.** SNP and InDel information.

**Additional file 3: Table S3.** Pairwise fixation index (*F*_ST_) values among 32 populations.

**Additional file 4: Table S4.** Gene number annoated from different databases.

**Additional file 5: Figure S1.** GO Enrichment analysis of marker-trait association related genes.

## Data Availability

The sequencing data generated in this study for the 223 samples is currently being submitted to the NCBI Sequence Read Archive (https://www.ncbi.nlm.nih.gov/sra) under the BioProject accession PRJNA924019 with run accession numbers from SRX19037395 to SRX19037617.
