# Supplementary figures and images for: The effect of environmental factors on the genetic differentiation of Cucurbita ficifolia populations based on whole-genome resequencing
Source: BMC Plant Biol. 2023 Dec 15;23:647. doi: 10.1186/s12870-023-04602-3 (PMC10722772; doi:10.1186/s12870-023-04602-3)

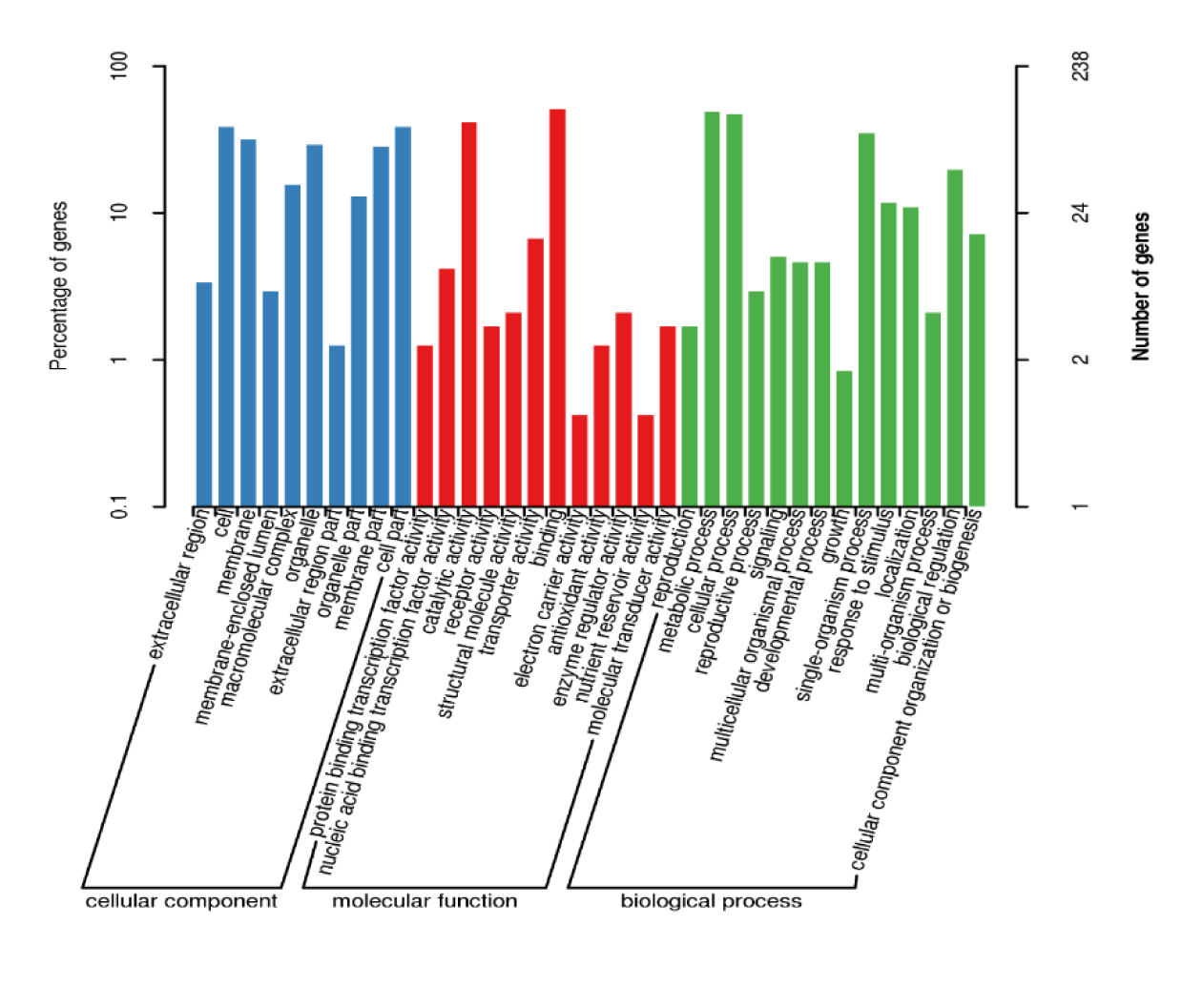

Supplement: Supplementary file 5 — Additional file 5: Figure S1. GO Enrichment analysis of marker-trait association related genes. [file 12870_2023_4602_MOESM5_ESM.tif]
